# Fracture resistance of prefabricated versus custom-made zirconia crowns after thermo-mechanical aging: an in-vitro study

**DOI:** 10.1186/s12903-022-02628-x

**Published:** 2022-12-09

**Authors:** Osama Ibrahim El Shahawy, Maha Moussa Azab

**Affiliations:** 1 Department of Pediatric Dentistry, Faculty of Dentistry, Cairo University, Cairo, Egypt; 2 Department of Pediatric Dentistry, Faculty of Dentistry, Fayoum University, Fayoum, Egypt; 3 Department of Pediatric Dentistry, School of Dentistry, Newgiza University NGU, Giza, Egypt

**Keywords:** Prefabricated crowns, Young permanent molars, Cercon ht, Thermo-mechanical aging

## Abstract

**Background:**

Prefabricated zirconia crowns for a young permanent molar is a child-friendly solution for restoring a permanent molar at a young age. This in-vitro study aimed to compare the fracture resistance of prefabricated versus custom-made permanent molar crowns.

**Methods:**

16 identical resin dies were fabricated to receive permanent molar zirconia crowns, dies were divided into 2 groups, 1) received perfricated crowns, 2) custom-made crowns. Thermo-dynamic cycling was performed to simulate 6 months in the oral cavity, Fracture resistance of each group was assessed by applying increasing load till fracture. Data were tested for normality using Shapiro–Wilk and Levene's tests. Data were analyzed using independent *t* test.

**Results:**

No statistically significant difference was found between fracture resistance of prefabricated and custom-made crowns (1793.54 ± 423.82) and (1987.38 ± 414.88) respectively. 3 crowns of the custom-made group fractured with the underlying die, versus zero dies fractured in the prefabricated group.

**Conclusions:**

Prefabricated permanent molars zirconia crowns can perform as well as custom-made crowns for an adult in terms of fracture resistance, it is suitable for children and can withstand the occlusal forces of an adult.

## Background

Dental decay is the most common chronic disease affecting children [1]. FPMs erupting at around six years of age are prone to decay at a very young age [2]. Moreover, FPM affected with MIH or other hereditary or developmental conditions may need serious restorative work at a very young age [3]. Restoration of young permanent teeth is a challenge, as the dentist should balance between the simplicity of the treatment delivered at this young age and delivering a definitive treatment to a permanent tooth.

The SSC as a full coverage restoration was considered for a long time as the gold standard for the restoration of children’s molars, restoring primary and permanent molars with developmental and inherited conditions, cervical caries and multiple surface caries with superior durability [4, 5].

Due to the continuous increase in socio-economic standards; restoring posterior teeth with esthetic restorations is increasingly in high demand. Translucent zirconia is now widely used as a dental restorative material due to its favourable biological, mechanical, and esthetic properties [6, 7].

The commonly used 3 mol% Y-TZP has been proven to have sufficient mechanical properties to withstand occlusal forces as single posterior crowns, some research work is testing its performance as a 3-unit posterior bridge, while the more translucent 4 and 5 mol % yttria are recommended for anterior restoration [8–10].

3 mol% Y-TZP custom-made zirconia crowns are prepared using CAD/CAM technology in a single-visit procedure; however, the multiple steps of teeth preparation, impression taking, milling and crown cementation, are too long and too complicated for a child, and this comes in addition to the necessary equipment, knowledge and training needed to complete this procedure [11].

Prefabricated zirconia crowns for children are available for primary incisors, primary molars and FPMs [12]. PZCpri are clinically successful, gingival friendly, esthetically pleasing and satisfying for parents and children [13]. PZCperm was found beneficial and promising in cases of early multiple surface caries, pulp treatment and malformed teeth as in MIH [14], PZCperm require extensive tooth preparation, come in limited shades and have standard anatomy which makes it challenging to fit into different teeth alignments and occlusions, it is on the pricy side of dental restorations as well; however, the easy and short time procedure make it a suitable restoration for this age group [15].

A very limited number of studies have dealt with PZCperm; Deeb et al. have studied the retrieval of cemented PZCpri and PZCperm crowns using Er,Cr:YSGG [16], Stepp et al. compared the microleakage of 2 brands of PZCperm when cemented with 2 different types of dental cements in-vitro [17].

However, PZCperm has not yet been tested to function under an adult's mastication forces and oral conditions, and the question of whether aged PZCperm would withstand the occlusal forces of an adult without fracture is yet to be answered.

Therefore, the aim of this in-vitro study was to compare the fracture resistance of prefabricated versus custom-made FPM crowns cemented to a resin FPM die after thermomechanical aging. The null hypothesis tested was that there is no difference in the fracture resistance between the 2 crowns.

## Methods

The study was approved by the Supreme Committee for Scientific Research Ethics (approval date: 4–22).

### Crown preparation

**Fig. 1:**
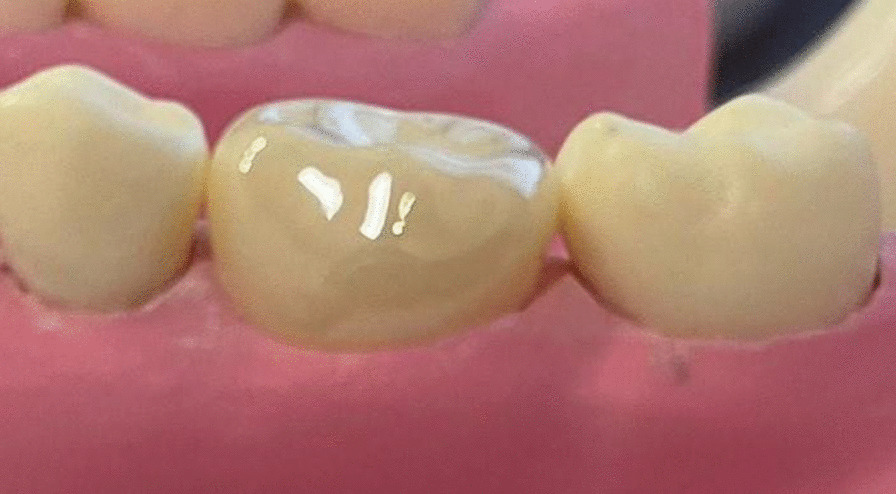
Fitted prefabricated zirconia crown on the prepared educational model

On a synthetic lower arch educational model (Banna dental simulation, Cairo, Egypt), the mandibular left FPM was prepared with a circumferential feather-edge finish line and rounded line and point angles, the preparation was adjusted to passively receive a suitable PZCperm (NuSmile®, Houston, Texas, USA), size 5 crown fitted the preparation properly, Fig. 1.

### Die fabrication

The prepared molar and adjacent teeth were digitally scanned using CEREC Omnicam (Sirona Dental Systems, Bensheim, Germany) to produce a 3D digital model.

16 resin FPM dies (NextDent Model 2.0 B.V. Centurionbaan, Soesterberg, The Netherlands) were produced from the digital model.

### Custom-made crowns

A custom-made zirconia crown was designed with an axial and occlusal walls thickness of 1.4 mm using (Exocad GmbH, Germany), then 8 identical zirconia crowns (Cercon ht (Dentsply Sirona, Bensheim, Germany) were milled out using (PrograMill PM7. Ivoclar digital. © Ivoclar Vivadent AG, Schaan/Liechtenstein). Sintering was done following the manufacturer’s recommendations, using the (Programat S1 1600. © Ivoclar Vivadent AG, Schaan/Liechtenstein) furnace. Crowns were then sandblasted.

**Fig. 2:**
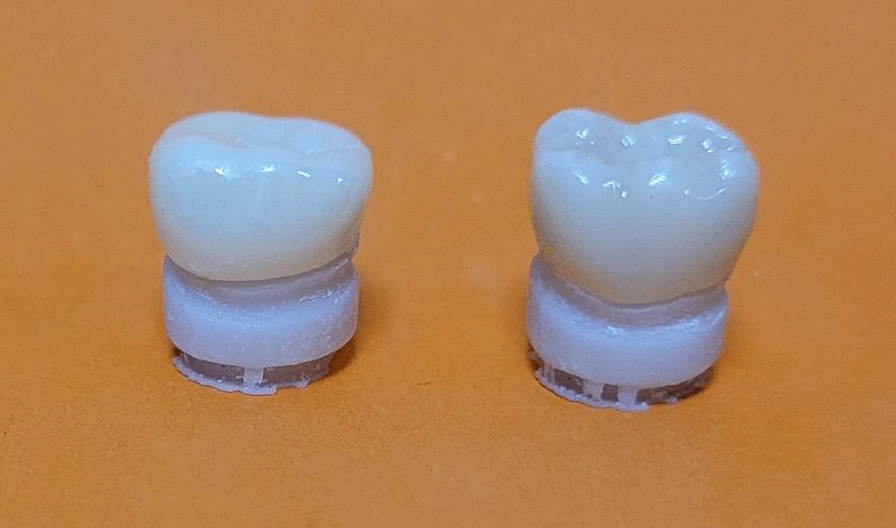
Prefabricated (on the left) and custom-made (on the right) zirconia crowns on corresponding dies

All the prefabricated and custom-made crowns were inspected for cracks, chipping and other defects under × 35 magnification (U500x Digital Microscope, Guangdong, China), crowns are shown in Fig. 2.

All 16 crowns were cemented to their respective dies using glass-ionomer cement (Medicem, Promedica Domagkstrasse, Neumuenster, Germany). Each crown was placed under a static load of 50 N for 5 min, excess cement was removed, and the crowns were placed in distilled water at 37◦C for 24 h.

### Thermo-dynamic cycling

To simulate 6 months in the oral cavity, crowns were subjected to 5000 thermal cycles of 5 °C–55 °C, with dwell time = 25 s, and lag time = 10 s using (Robota automated thermal cycle; BILGE, Turkey) [18]. Then a chewing simulator (ROBOTA, Model ACH-09075DC-T, AD-TECH TECHNOLOGY CO., LTD., GERMANY) was used to apply 75,000 cycles of 50 N occlusal load at 1.6 Hz frequency [19].

### Fracture resistance

The occlusal surface of each crown was loaded with a metallic rod with a spherical tip (5.8 mm diameter) using a computer-controlled testing machine (Model 3345; Instron Industrial Products, Norwood, MA, USA) at 1 mm/min until fracture. The spherical tip was cushioned with a standardized tin foil sheet for homogenous load distribution, and to avoid local damage during loading. Failure load was determined at the first audible crack, confirmed by a drop in the load–deflection curve, data were recorded using computer software (Bluehill Lite Software, Instron®).

Each crown was inspected using a microscope to assess their fracture mode, crowns were classified according to Burke’s classification for modes of fracture [20], Table 1.

**Table 1:** Burke’s classification for modes of crown fracture

Code	Interpretation
I	Minimal fracture or crack in crown
II	Less than half of crown lost
III	Crown fracture through midline
IV	More than half of crown lost
V	Severe fracture of crown and/or tooth

### Statistical analysis

Categorical data were presented as frequency and percentage values and were analyzed using chi-square test followed by pairwise comparisons utilizing multiple z-tests with Bonferroni correction. Numerical data were represented as mean and standard deviation (SD) values. They were tested for normality and variance homogeneity using Shapiro–Wilk and Levene's tests respectively. Data were normally distributed and showed variance homogeneity across tested groups. They were analyzed using an independent *t* test. The significance level was set at *p* < 0.05 within all tests. Statistical analysis was performed with R statistical analysis software version 4.1.3 for Windows [21].

## Results

**Fig. 3:**
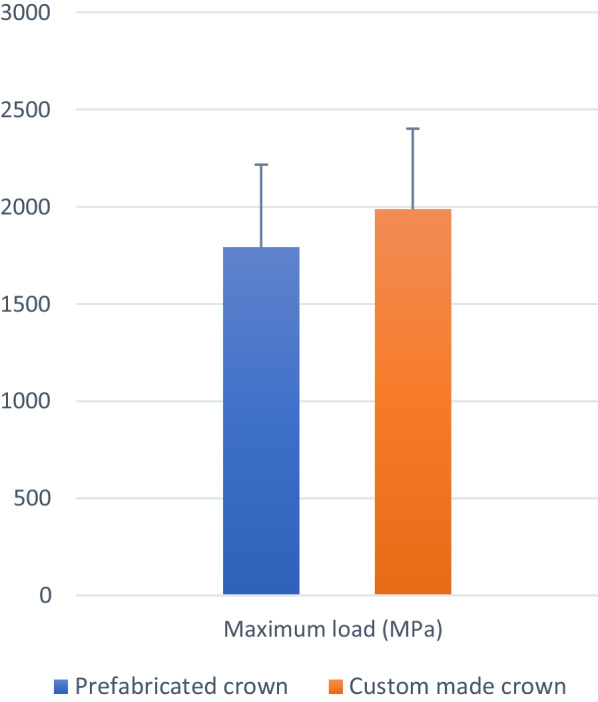
Bar chart showing mean and standard deviation values of fracture  loads (N) in different groups

Results of intergroup comparisons presented in Table 2, showed no statistically significant difference between fracture resistance of custom-made or prefabricated Zirconia crowns (t = 0.92, *p* = 0.371). However, it was slightly higher for custom-made crowns (1987.38 ± 414.88) than for prefabricated ones (1793.54 ± 423.82). Mean and standard deviation values of fracture loads in different groups were presented in Fig. 3.

**Table 2:** Intergroup comparison of fracture strength

Maximum load (MPa) (Mean ± SD)		Mean difference [95% CI)	t-value	*p*-value
Prefabricated crowns	Custom made crowns			
1793.54 ± 423.82	1987.38 ± 414.88	− 193.84 [− 643.57: 255.89]	**0.92**	**0.371**

**Fig. 4:**
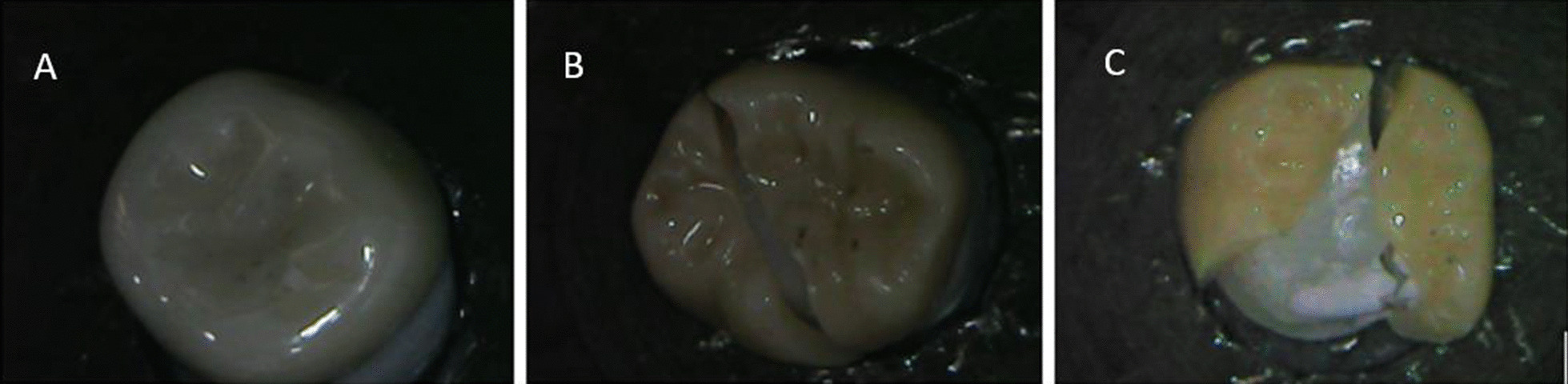
Examples of fractured specimens. **A** Class I (crack in crown). **B** Class III (crown fracture through midline). **C** Class V, Catastrophic mode (severe fracture of the crown and tooth)

Results of intergroup comparisons of failure mode presented in Table 3, showed that there was a statistically significant higher percentage of code (II) fracture mode in the custom-made crowns group (χ^2^ = 12.80, *p* = 0.012. Examples of fractured specimens are presented in Fig. 4

**Table 3:** Intergroup comparison of failure mode

Failure mode		Prefabricated crown	Custom made crown	χ^2^	*p*-value
I	n	1	0	**12.80**	0.012*
	%	12.5%	0.0%		
II	n	0	4*		
	%	0.0%	50.0%		
III	n	4	1		
	%	50.0%	12.5%		
IV	n	3	0		
	%	37.5%	0.0%		
V	n	0	3		
	%	0.0%	37.5%		

*Significant (*p* < 0.05)

## Discussion

This study is the first to address the fracture resistance of PZCperm in comparison to custom-made zirconia crowns, fracture resistance is one of the parameters to determine the survival of restoration and its ability to withstand occlusal forces. PZCperm are usually used at young ages, taking advantage of the easy application, short chair side time, and being superior to the SSC for permanent molars in esthetics and biocompatibility [22].

In this study, crown preparation ended with a feather edge finish line compatible with PZCperm, feather edge finish line is recently introduced and found suitable for custom-made zirconia crowns as well [23, 24].

We used the same die for all samples to avoid any variation between the crowns of the two study groups. 3D-printed dies were chosen to standardize dies for all samples. The use of natural teeth dies was not practical due to standardization difficulties as they come with different ages, storage conditions, shapes, and sizes. The selected die material (NextDent Model 2.0 B.V. Centurionbaan, Soesterberg, The Netherlands) was used in a previous study by Kongkiatkamon et al., it was chosen because it has mechanical properties close to that of enamel and dentine [25, 26]

Thermal and mechanical cycling was done to produce aging of ceramic restorations, to simulate aging in the oral cavity, this is used in in-vitro studies to develop subcritical crack growth, creating a condition close to that existing in reality [27].

To evaluate the fracture resistance of PZCperm, we compared it to previously tested and widely used custom-made zirconia crowns Cercon ht [10, 25, 28].

The fracture resistance results of the current study showed that there was no statistical difference between prefabricated crowns and custom-made ones, therefore, the study’s null hypothesis is accepted. Several previous studies have studied the fracture load of Cercon ht; in Nejat et al., the fracture load for Cercon ht crowns was significantly higher than that for Cercon xt crowns with the same occlusal thickness [28]. Kongkiatkamon et al., found that Cercon ht had a significantly higher fracture load than AmannGirrbach, Cercon xt, and Vita ZY XT [25].

PZCperm were designed to deliver acceptable mechanical properties and esthetics with the simplest application procedure suitable for the younger age group; challenging a lot of the custom-made crowns application postulates as solid margins and finish lines, close fit, minimal and uniform cement space ……, all of the aforementioned factors can affect the mechanical properties and behaviour of PZCperm on function; however, from the current study and previous work results, we can conclude that PZCperm can withstand occlusal loads comparable to Cercon ht, which is one of the superior, widely used zirconia materials. Therefore, in terms of strength and fracture resistance, PZCperm can perform well in the oral environment of an adult. It is worth mentioning that the mean posterior bite force is ± 850 N, which is well below the fracture loads recorded for both groups in the current study [29].

Burke’s classification was used by previous studies to classify fracture mode [30] In the current study custom-made crowns group had statistically significant more code II fracture mode (fracture where less than half of the crown lost). It is worth mentioning that for the custom-made group, 3 out of the 8 samples fractured with code V (Severe fracture of crown and/or tooth = catastrophic failure mode), the case was different with the prefabricated crowns group where no dies have fractured with the crowns. Hassouneh et al. and Zarone et al. suggested that catastrophic fractures might be due to the high values of fracture load. In our study, this was the case with the custom-made group which had a higher mean fracture load than the other group [31, 32].

However, this study has some limitations, being an in-vitro study, and being the first to compare between PZCperm and custom-made crowns, there was no previous work to estimate sample size. More studies with larger sample sizes and a longer aging period can add to the data concluded from the current study, also, in-vivo trials testing the clinical performance of the PZCperm regarding fracture resistance and other parameters such as biocompatibility, occlusion development, and the effect of apical migration of gingival margin on PZCperm would help to answer the question of whether we need to replace PZCperm at some point or not.

## Conclusions

From the results of the given study, one can conclude that Nusmile PZCperm can perform as well as custom-made Cercon ht zirconia crowns for an adult, in terms of fracture resistance, the fracture load of PZCperm is well above the documented maximum occlusal loads. With the advantages of short chair-side time, easy application and simple procedure; PZCperm originally recommended for young FPM at young ages where child cooperation is questionable, is a durable restoration that can withstand the occlusal forces of an adult.

## Data Availability

All data generated or analysed during this study are included in this published article.

## Abbreviations

FPM: First permanent molars
MIH: Molar incisor hypomineralization
SSC: Stainless-steel crown
Y-TZP: Yttria-stabilized tetragonal zirconia polycrystal
PZCpri: Prefabricated zirconia crowns for primary teeth
PZCperm: Prefabricated zirconia crowns for permanent first molars
